# Early Biomedical and Environmental Factors Associated with Developmental Coordination Disorder in a Brazilian Preterm Cohort

**DOI:** 10.3390/brainsci15121250

**Published:** 2025-11-21

**Authors:** Carolina Panceri, Renato Soibelmann Procianoy, Rita de Cássia Silveira, Nadia Cristina Valentini

**Affiliations:** 1 Hospital de Clínicas de Porto Alegre, Porto Alegre 90035-903, Brazil; carolpanceri@hotmail.com (C.P.); rprocianoy@gmail.com (R.S.P.); drarita.c.s@gmail.com (R.d.C.S.); 2 Department of Physical Education, Physiotherapy and Dance, Universidade Federal do Rio Grande do Sul, Porto Alegre 90690-200, Brazil

**Keywords:** child development, preterm, risk factors, motor skills disorder, developmental coordination disorder, neurodevelopment

## Abstract

Background: Developmental Coordination Disorder (DCD) is frequent in preterm children, but its etiology remains unclear, with conflicting findings across studies. Moreover, no previous research has examined the rate and risk factors of DCD in Brazilian preterm populations. Aim: To investigate biomedical and environmental risk factors associated with a later indication of DCD at preschool age in Brazilian preterm children. Methods: Sixty-three preterm children from a follow-up clinic were assessed at preschool age using the Movement Assessment Battery for Children—Second Edition (MABC-2). Biomedical data from the NICU stay and socioeconomic/environmental information were collected. The associations between potential risk factors and DCD were explored using multivariate logistic regression. Results: The prevalence of DCD was 52.4% (n = 33). Group differences were observed in sex, bronchopulmonary dysplasia, ventilatory support, and family income. Logistic regression [χ^2^(4) = 31.39; *p* < 0.006] identified sex, bronchopulmonary dysplasia, and family income as significantly factors associated with DCD. Conclusions: Both biomedical and environmental risk factors are associated with DCD at preschool age. These findings highlight the need for early screening and monitoring, as minor motor difficulties may be overlooked when such risk factors are observed.

## 1. Introduction

Developmental coordination disorder (DCD) is observed in an estimate of 5–6% of school-aged children worldwide [1], with considerably higher prevalence among preterm children, ranging from 9 to 48% [2]. DCD is characterized by poor motor coordination that interferes with daily functioning at school, as well as during play, leisure, and self-care, and cannot be explained by intellectual or visual impairment, or other neurological conditions [3]. Early delays in motor milestones are frequently reported, and difficulties may persist into adolescence [4] and adulthood [1], with broad implications for academic achievement [5], social participation [4,6], health [7], and quality of life.

Preterm children are at substantially greater risk of developing DCD than their full-term peers [8,9,10], and several biomedical and environmental risk factors have been implicated, including male sex [11], bronchopulmonary dysplasia and chronic lung disease [12], retinopathy of prematurity [11], smaller brain volumes [13], and perinatal complications [14]. Environmental factors such as low parental education, socioeconomic disadvantage, and exposure to tobacco during pregnancy have also been reported [9,14]. However, findings across studies remain inconsistent, and most evidence comes from high-income countries [10].

Research on DCD has been disproportionately concentrated in WEIRD (Western, Educated, Industrialized, Rich, and Democratic) contexts over the past two decades, led by Canada, the UK, Australia, the USA, and the Netherlands [10,15]. Very few studies originate from low- and middle-income countries (LMICs), where children are often more exposed to poverty-related risks such as inadequate prenatal care, poor maternal education, nutritional deprivation, and limited stimulation at home [16]. In Brazil, the available studies on DCD are limited to the general population, with no investigation to date of risk factors in preterm cohorts. This represents a critical gap, given that preterm children in LMICs face unique biomedical and environmental challenges that may shape developmental outcomes differently than in high-income contexts.

Investigating early biomedical and environmental predictors of DCD in LMIC preterm populations is essential to improving early detection and guiding preventive interventions. Therefore, this study aimed to examine the association between early biomedical and environmental risk factors that are indicative of later DCD diagnosis at preschool age in a cohort of Brazilian preterm children. It is hypothesized that biomedical and environmental risk factors in early life are linked to a higher risk of DCD at preschool age in Brazilian preterm children.

## 2. Materials and Methods

### 2.1. Context and Participants

**Figure 1:**
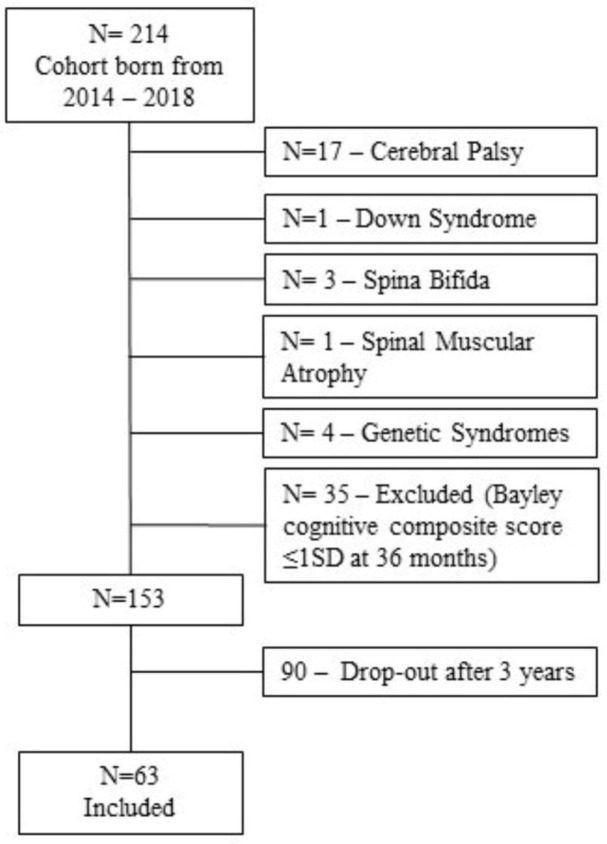
Participants’ enrollment across the study.

The study adhered to international ethical standards, specifically the Declaration of Helsinki, and was carried out in accordance with the principles of Resolution 466/12 of the National Health Council. The research received formal approval from the local hospital Institutional Ethics Committee (process nº 20190321) for studies involving humans. The children included in the present study were born from May 2014 to December 2018 and are part of a cohort study conducted in a Brazilian public hospital. All inborn preterm infants whose gestational age was less than 32 weeks and/or whose birth weight was less than 1500 g are designated to attend the Neonatology Outpatient Clinic. They have frequent scheduled follow-up multidisciplinary appointments until 5 years of age, following the hospital’s standard care process. The exclusion criteria for this study were children with cerebral palsy, congenital malformation, and genetic syndromes. Sixty-three preschool children participated (M_age_ = 52.10 months, SD 9.66). Figure 1 presents a flow chart of participants’ enrollment across the study. The study was designed and reported according to the STROBE (Strengthening the Reporting of Observational Studies in Epidemiology) checklist for cohort studies to ensure methodological transparency and rigor.

### 2.2. Classification of DCD

DCD is diagnosed using four criteria established by the Diagnostic and Statistical Manual of Mental Disorders (DSM-V), 5th edition: (1) motor coordination performance is significantly lower than expected for age; (2) motor difficulties impact daily activities and school performance; (3) the onset of motor difficulties is observed in the early developmental period; (4) motor coordination difficulties are not explained by intellectual impairment, visual impairment, or other neurological conditions [3].

The Movement Assessment Battery for Children, second edition (MABC-2) [17] was applied to assess children’s motor coordination, consistent with the first DSM-V recommendation and the European Academy of Childhood Disability’s guidelines [1,3]. This is the most common tool used to detect motor coordination difficulties in children with DCD, including children born preterm. In this study, children who scored in the ≤5th percentile on the MABC-2 were identified as having DCD, and children who scored in the ≥16th percentile were identified as displaying typical motor development, consistent with other studies [14,18]. Children who scored in the “at risk” category (within the 6th and 15th percentiles) were identified as “non-DCD”, since these children did not meet all DSM-V criteria, and children who scored in the ≥16th percentile on the MABC-2 were also classified as “non-DCD”. Although some authors use the 15th percentile as the cut-off criterion [19], a more conservative cut-off (≤5th percentile) was applied, aligning with the recommendations for children aged 3–5 years [20].

The impact of motor difficulties on the child’s life was measured by structured parent interviews in the follow-up appointments, considering the second recommendation of the DSM-V [1,3]. For the third DSM-V criterion, the Bayley Scales of Infant and Toddler Development third edition (BSITD-III) [20] was repeatedly applied during the first 3 years of life, helping to detect impairments that may be causal and those that may co-occur [1,3]. Regarding the fourth DSM-V criteria, the medical team in the follow-up clinic provided the diagnosis of visual impairment, cerebral palsy, or other neurological conditions. In addition, we excluded children who had cognitive impairment at 3 years of age (BSITD-III composite score ≤ 1 SD).

### 2.3. Assessments

Children’s biomedical factors were collected from neonatal intensive care unit (NICU) records (i.e., gestational age, birth length and weight, head circumference, APGAR 5th, ventilator support, necrotizing enterocolitis, intraventricular hemorrhage, periventricular leukomalacia, parenteral nutrition, bronchopulmonary dysplasia, sepsis), as well as mother’s biomedical factors (i.e., antenatal steroids use, prenatal appointments, pre-eclampsia).

Standardized clinical procedures were used to ensure the reliability of data. Intraventricular hemorrhage was diagnosed through weekly cranial ultrasonography until the sixth week of life or hospital discharge. For periventricular leukomalacia, the initial diagnosis relied on cranial ultrasonography and was confirmed through magnetic resonance imaging of the brain at term equivalent, before hospital discharge. Ventilatory support was analyzed by days (mechanical ventilation plus non-invasive ventilation), and the Apgar score ranged from 0 to 10. Bronchopulmonary dysplasia (BPD) was diagnosed as the child needing oxygen for ≥28 days of life, sepsis was diagnosed using positive blood culture with the presence of clinical signs of infection, and enterocolitis was diagnosed by the presence of pneumatosis intestinalis and/or pneumoperitoneum. During the first year of life, on the follow-up visits at 4, 8, and 12 months of corrected age and at 36 months of chronological age, parents were asked to report on children’s signs of progress, difficulties in their daily routine, nutrition, and general health concerns.

Movement Assessment Battery for Children, second edition (MABC-2) [17] was applied to evaluate whether Developmental Coordination Disorder was present between 3 and 6 years old. The MABC-2 assesses manual dexterity, ball skills, and static and dynamic balance, and it was validated for Brazilian children using a representative sample [21,22]. The standard score is derived from raw scores, and the sum of the standard scores provides the Total Motor Impairment Score, which is converted into a percentile. The MABC-2 manual suggests that children who score in the 6th–15th percentile are “at-risk” of having a movement difficulty, whereas children who score in the ≤5th percentile have a significant movement difficulty [17].

The present study classified DCD when the MABC-2 percentile was ≤ 5%. The ≤5% cut-off is consistent with other studies [13,17] and was adopted since the difficulties experienced by these children are more likely to interfere in their daily life [17]. The DSM-V recommendations were followed to complete the child’s DCD diagnosis. Therefore, if the child showed difficulties in daily life at home and school (reported by parents during consultations), had an absence of neurological conditions (reported by the pediatric neurologist at the follow-up clinic), and showed delays in motor milestones (assessed by BSITD-III in the follow-up clinic in the first year), they were categorized as having DCD.

The Bayley Scales of Infant and Toddler Development third edition (BSITD-III) [21] was applied to assess the early onset of motor difficulties (at 4, 8, and 12 months of corrected age) and cognitive development at 36 months. The BSITD-III composite scores have a standardized mean of 100 with standard deviations (SDs) of 15 points. Following the recommended guidelines, impairment categorization was identified if the child scored ≤1 SD [21]. For the present study, we excluded children who scored ≤1 SD in cognitive assessment at 36 months of age.

During the scheduled follow-up appointments, parents were asked to complete a survey designed to gather information about the family’s socioeconomic status and environmental factors, including family income and the level of formal education for both parents. The parents’ level of formal education was categorized as (1) less than high school, (2) completed high school, and (3) college and/or university; family income was considered as the sum of the monthly income, in Brazilian currency, of all the people who live in the residence.

Three additional instruments were used to characterize the caregiving and home environment. The Knowledge of Infant Development Inventory (KIDI) [23] has been validate for the Portuguese language; the results confirmed a unidimensional structure and revealed good internal consistency (Cronbach’s α = 0.89). Ref. [23] used KIDI at 4 months to assess parental knowledge regarding infant development. For this age group, KIDI comprises 20 items that specifically ask about the age at which infants typically acquire certain skills. The total score is calculated as the ratio between the number of correct answers and the total number of items (1 is the maximum score) [23].

The Interaction Rating Scale (IRS) was applied at 12 months to evaluate the mother/child duos. The IRS is an observational tool designed to assess key aspects of caregiving and child behavior, including a child’s social skills, the caregiver’s parenting skills, and the caregiver–child interactions [24]. The scale is composed of 70 dichotomous items (1 = yes; 0 = no), and the overall observed behavior score is determined by summing the score of all these items. Since the instrument has not yet been validated for the Brazilian population, a validation procedure was conducted within the present sample. The IRS showed strong internal consistency (Cronbach’s α = 0.93) and high inter-rater (96% agreement) and intra-rater (97% of agreement) reliability. These indicators support the adequacy of the instrument for assessing this specific population.

The Affordance in the Home Environment for Motor Development—Infant Scale (AHEMD-IS) was administered at 12 months of corrected age. This scale aims to evaluate developmental opportunities present in the child’s home environment, specifically examining factors related to physical space, daily activities (both outside and inside), and available play materials. The scale provides both a total score and subsequent categorization (less than adequate: 0 to 18; moderately adequate: 19 to 23; adequate: 24 to 27; excellent: 28 to 49) [25].

### 2.4. Procedures

The Neonatology Outpatient Clinic’s standard follow-up protocol includes systematic BSITD-III assessment from birth to 36 months of chronological age. An additional MABC-2 assessment was incorporated between 3 and 6 years to prospectively identify DCD cases.

Questionnaires were administered at pre-established time points (sociodemographic and KIDI at 4 months of corrected age; AHEMD-IS and IRS at 12 months). Two experienced professionals (each with over five years of experience in child development) conducted all assessments independently. Inter-rater reliability was high (96% agreement).

### 2.5. Data Analyses

The sample size calculation was based on a minimum of five events per predictor variable entering the multivariate logistic regression, since several confounding factors were controlled for in the present study [26]. The general recommendation of at least five observations per independent variable is supported in the literature [27].

Given that both maternal and child biomedical and environmental risk factors were included, we adopted a rule of a minimum of five events per predictor and a maximum of twelve predictors: sex, gestational age, birth weight, length of NICU stay, ventilatory support, bronchopulmonary dysplasia, sepsis, seizures, maternal age at child’s birth, paternal age at child’s birth, family income, and maternal formal education. Based on this criterion, the required minimum sample size was 60 children [26].

Descriptive analyses were conducted using IBM SPSS Statistics, version 20. Means, standard deviations, and frequencies were estimated for the study variables. Group differences between children with and without DCD were examined using Student’s *t*-test for continuous variables and the Chi-square test for categorical variables.

To explore the associations between potential risk factors and DCD, a multivariate logistic regression analysis was performed. All independent variables (sex, gestational age, birth weight, NICU stay, ventilatory support, bronchopulmonary dysplasia, sepsis, seizures, maternal age at child’s birth, paternal age at child’s birth, family income, and maternal education) were first tested in univariate logistic regressions. Variables with *p* ≤ 0.05 were entered into the multivariate logistic model. Collinearity between variables was examined prior to model entry to ensure statistical validity.

## 3. Results

The analysis included 63 children. A comparison with the 90 dropouts—most of whom withdrew in the early years of the study due to the COVID-19 pandemic—revealed no statistically significant differences across biomedical characteristics, environmental variable, or BSITD-III scores. *t*-test showed *p* = 0.374 for gestational age, *p* = 0.851 for birth weight, *p* = 0.842 for birth length, *p* = 0.935 for head circumference, *p* = 0.069 for APGAR 5th minute, *p* = 0.672 for days in Neonatal Intensive Care Unit, *p* = 0.346 for ventilatory support, *p* = 0.091 for mother’s age, *p* =0.100 for father’s age, and *p* = 0.584 for family income. Chi-square teste showed *p* = 0.194 for bronchopulmonary dysplasia, *p* = 0.246 for sepsis, *p* = 0.484 for necrotizing enterocolitis, *p* = 0.395 for intraventricular hemorrhage, *p* = 0.256 for periventricular leukomalacia, *p* = 0.481 for mother’s formal education, and *p* = 0.445 for father’s formal education. Bayley scores for the cognition (*p* = 0.330), language (*p* = 0.901), and motor (*p* = 0.110) domains were also not significant.

The mean age at MABC-2 assessment was 52.10 months (SD 9.66), and the DCD prevalence among the cohort was 52.38% (N = 33) at preschool age. Table 1 presents the biomedical risk factors of children and maternal health characteristics for each group and the total sample. Significant group differences were found; a higher proportion of boys were diagnosed with DCD, children with DCD required more days in ventilatory support after birth and had higher prevalence of bronchopulmonary dysplasia. The variables that reached significance in the univariate logistic analysis were subsequently entered into the multivariate model.

Environmental factors potentially influencing child development are presented in Table 2. No significant differences were found for maternal knowledge regarding infant development, mother–child interaction, and home affordances for motor development. However, families of children with DCD reported significantly lower household incomes. Family income also remained significant in the univariate logistic regression and was entered into the multivariate model.

Univariate logistic regressions analyses indicated sex, ventilatory support, bronchopulmonary dysplasia, and family income associated with DCD. The multivariate logistic regression model incorporating these variables was statistically significant [X^2^(4) = 31.391; *p* < 0.006]. The final model showed that sex, the presence of bronchopulmonary dysplasia, and family income were the variables that remained significantly associated with DCD. The overall goodness-of-fit of the logistic regression model was assessed using the Hosmer–Lemeshow test, which yielded a non-significant *p*-value (*p* = 0.929), indicating excellent model calibration. The model correctly classified approximately 81% of the cases. Table 3 presents the results of the multivariate logistic regression model.

In summary, being a boy and having a history of bronchopulmonary dysplasia were significant factors associated with a higher likelihood of being diagnosed with DCD. In contrast, higher family income was significantly associated with a lower likelihood of the disorder among the children studied. These findings highlight the combined influence of neonatal complications and socioeconomic conditions on later motor outcomes in preschool-aged children born preterm.

## 4. Discussion

This study examined the differences between DCD and non-DCD preterm groups and the potential risk factors associated with DCD. Our findings revealed that more than half of the sample (52.38%) of very and extremely preterm children met the DSM-V criteria for DCD, a rate that is notably higher than that previously reported in the international literature for preterm children [1,10]. However, this finding is consistent with the rate of 51.72% reported by Deshmukh, Sahu, and Deshpande (2024) in their analysis of both premature and low-birth-weight children [28]. This elevated prevalence in this cohort underscores the potential socioeconomic vulnerability of preterm children in low- and middle-income contexts and reinforces the urgent need for early identification and intervention.

The comparison of children with and without DCD revealed significant differences in sex, length of ventilatory support, prevalence of bronchopulmonary dysplasia, and family income. DCD was more prevalent in boys than girls; children with DCD required longer ventilatory support, were more likely to develop bronchopulmonary dysplasia, and tended to belong to families with lower income levels.

Importantly, the multivariate logistic regression model confirmed that sex, bronchopulmonary dysplasia, and family income remained significantly associated with DCD, whereas ventilatory support lost significance after adjusting for confounders. Our findings were consistent with previous studies reporting that DCD occurs more frequently in preterm boys [8,11,14]. This male predominance appears robust across populations [1], with reported male-to-female ratios ranging from 2:1 to 7:1, although some studies failed to replicate this difference [18].

Respiratory morbidities emerged as a key biomedical factor. Conditions such as bronchopulmonary dysplasia (BPD), chronic lung disease, or the need for prolonged ventilatory support have been linked not only to DCD [8,12,18,29] but also to other subtle motor impairments in preterm populations [13,18,29]. In the present study, bronchopulmonary dysplasia stood out as the most powerful associated factor with DCD, even after adjusting for other neonatal and socioeconomic variables. Although some studies have reported contradictory results [13,18], the preponderance of evidence supports that neonatal hypoxia and oxygen instability can contribute to neurodevelopmental vulnerabilities, particularly affecting the brain regions involved in motor coordination [12,29,30]. Our data add to this evidence by showing that this association persists even in a low-resource healthcare context, where respiratory complications remain common.

The lack of significant differences in the proximal environmental measures (KIDI, IRS, AHEMD-IS) is important as this suggests relative homogeneity in the sample concerning these specific aspects of the caregiving environment. Furthermore, these results show that our cohort was predominantly composed of families from disadvantaged contexts. Nevertheless, our results reinforce the contribution of socioeconomic disadvantage to the persistence of DCD symptoms. Family income remained a significant explanatory variable, albeit with modest statistical association. This aligns with findings that children from higher socioeconomic backgrounds or with more educated parents are less likely to present with DCD [11,14]. Socioeconomic status influences not only access to healthcare and early intervention but also the quality of environmental affordances for motor development—such as play space, stimulation, and parental knowledge [31]. In our cohort, the predominance of families from disadvantaged contexts likely reduced variability in SES, possibly attenuating its statistical weight. Nevertheless, the persistence of family income as a predictor highlights the interplay between biomedical vulnerabilities and environmental constraints.

Interestingly, gestational age did not emerge as a significant factor associated with DCD. Although population-based studies have identified gestational age as a strong predictor of motor difficulties when the full spectrum of prematurity is considered [32], this association tends to diminish in homogeneous samples of very and extremely preterm children, such as ours [11,13]. The finding that gestational age did not emerge as a significant risk factor of DCD supports the view that supports that among very and extremely preterm infants, postnatal complications and socioeconomic environment may play a more decisive role in shaping later motor outcomes than gestational age itself.

DCD is often regarded as a “mild” motor impairment, yet its functional consequences are far from trivial, affecting academic achievement, social participation, and overall quality of life [6]. Our study advances understanding of DCD by examining both biomedical and environmental correlations within a low- and middle-income country (LMIC) context, where such data remain extremely limited. Clinicians and parents frequently focus on more severe conditions like cerebral palsy, risking under-recognition of DCD and, consequently, missed opportunities for timely intervention. Our results suggest that even in the absence of major neurological disorders, preterm children showing these risk factors should be systematically monitored for motor coordination difficulties.

The present study contributes novel evidence from Brazil, marking the first investigation of DCD risk factors in preterm children within this national context. The findings expand the global literature by highlighting the discussion on how the intersection of neonatal complications and socioeconomic adversity uniquely shapes neurodevelopment in LMIC settings. Furthermore, this study reinforces the crucial importance of a systematic assessment of motor development in premature infants. An early and thorough assessment by both clinicians and researchers is essential for detecting the full spectrum of motor impairments, which range from subtle to severe. Identifying these impairments as early as possible facilitates immediate referral for necessary interventions, including physiotherapy, adapted physical education, occupational therapy, or speech therapy.

### Strengths and Limitations

A major strength of this study lies in its longitudinal design, systematic follow-up, and simultaneous examination of multiple biomedical and environmental variables. Few studies have investigated such a comprehensive range of factors in preterm populations, particularly in countries with limited research infrastructure. However, the modest sample size and absence of a full-term control group limit generalizability. It is important to emphasize that these findings are derived from a cohort of very and extremely preterm children followed in a specific Brazilian public hospital system, a population known to face high biomedical and socioeconomic vulnerability. Consequently, these results cannot be generalized to the broader preterm population or to full-term children, but instead provide critical, context-specific evidence relevant to similar high-risk preterm cohorts in low- and middle-income countries.

Additionally, assessing DCD at preschool age may underestimate or overestimate prevalence, as motor difficulties may evolve during later childhood [2]. Future studies should pursue larger multicenter samples and longer follow-up into school years to confirm these findings.

## 5. Conclusions

This study provides the first evidence from Brazil—and is one of the few focusing on low- and middle-income countries (LMICs)—identifying an association between early biomedical and environmental risk factors and later indications of DCD in preterm children.

Our findings showed a high prevalence of DCD (52.38%) among very and extremely preterm children in this cohort and highlight that sex (male), bronchopulmonary dysplasia, and lower family income were factors significantly associated with of this condition. These results underscore the multifactorial nature of DCD, where biological vulnerabilities interact with socioeconomic constraints to shape neurodevelopmental outcomes.

Bronchopulmonary dysplasia emerged as the strongest biomedical factor associated with DCD, suggesting substantial association between early respiratory complications and long-term motor outcomes. At the same time, family income remained significant, reinforcing that environmental disadvantages can exacerbate developmental risks even when biomedical care is standardized.

Clinically, these findings call for the inclusion of DCD risk screenings in the routine follow-up of preterm children—especially boys, those with a history of respiratory complications, and those from socioeconomically vulnerable families. Early identification may allow interventions to be implemented before school entry, potentially minimizing the impact of DCD on academic, social, and daily functioning.

From a public health perspective, this study highlights the importance of strengthening early developmental surveillance within Brazil’s public health system and underscores the resilience and relevance of research conducted in public universities with limited resources.

Future studies should build on this work by expanding sample sizes, including full-term control groups, and following children longitudinally throughout school age to monitor developmental trajectories. Such research will contribute to a deeper understanding of how biomedical and social determinants intersect to shape motor development in diverse socioeconomic contexts.

## Figures and Tables

**Table 1:** Comparison and univariate logistic regression of mother and child biomedical factors between groups of DCD and non-DCD children and total sample.

Biomedical Characteristics			*t*-Test		Univariate Logistic Regression	
	DCD (n = 33)	Non-DCD (n = 30)	*p*	Total (n = 63)	OR (95% CI)	*p*
Child’s Biomedical Outcomes						
Sex N(%)						
Boys	26 (78.8)	11(36.7)	**0.001**	37 (58.7)	0.156 (0.048–0.458)	**0.001**
Girls	7 (21.2)	19 (63.3)		26 (41.3)		
Gestational age—weeks M (SD)	29.16 (2.56)	30.13 (2.19)	0.115	29.62 (2.42)	1.189 (0.963–1.496)	0.118
Birth weight—grams M (SD)	1226.00 (440.54)	1311.36 (361.90)	0.407	1266.64 (404.08)	1.001 (0.999–1.002)	0.401
Birth length—cm M (SD)	37.59 (4.08)	38.41 (3.65)	0.403	37.98 (3.88)	1.058 (0.930–1.210)	0.394
Head circumference—cm M (SD)	26.74 (3.35)	27.03 (2.39)	0.695	26.88 (2.91)	1.035 (0.872–1.233)	0.693
APGAR 5th minute M (SD)	7.15 (2.10)	7.80 (1.24)	0.139	7.46 (1.76)	1.252 (0.934–1.749)	0.136
NICU (days) M (SD)	73.85 (40.10)	57.53 (25.11)	0.056	66.08 (34.53)	0.985 (0.969–1.000)	0.066
Ventilatory support (days) M (SD)	33.75 (36.13)	13.20 (16.02)	**0.006**	52.76 (16.27)	0.969 (0.941–0.991)	**0.017**
Bronchopulmonary dysplasia N(%)	13 (39.4)	3 (10.0)	**0.010**	16 (25.4)	5.850 (1.628–28.051)	**0.012**
Sepsis N(%)	27 (81.8)	23 (76.7)	0.525	50 (79.4)	1.704 (0.481–6.442)	0.412
Necrotizing enterocolitis N (%)	3 (9.1)	0	0.119	3 (4.8)	--	--
Intraventricular hemorrhage N (%)						
Grade 0	20 (60.6)	24 (80.0)	0.154	44 (69.8)		
Grades 1 and 2	11 (33.3)	6 (20.0)		17 (26.9)	--	--
Grades 3 and 4	2 (6.1)	0		2 (3.2)		
Periventricular leukomalacia N (%)	0	2 (6.7)	0.216	2 (3.2)		
Parenteral nutrition M (SD)	16.18 (14.86)	13.83 (11.02)	0.483	15.06 (13.12)	0.986 (0.946–1.024)	0.472
Mother’s health						
Antenatal steroids N (%)	30 (90.9)	30 (100)	0.244	60 (95.2)	-	-
Prenatal appointments M (SD)	4.88 (2.45)	5.30 (1.51)	0.422	5.08 (2.05)	1.108 (0.868–1.439)	0.412
Pre-eclampsia N (%)	12 (36.4)	10 (33.3)	0.999	22 (34.9)	0.875 (0.305–2.475)	0.801

Note: M: Mean; SD Standard Deviation; N: Number; NICU: Neonatal Intensive Care Unit; significant results are presented in bold.

**Table 2:** Comparison and univariate logistic regression of environmental factors between groups of DCD and non-DCD children and total sample.

Biomedical Characteristics			*t*-Test		Univariate Logistic Regression	
	DCD (n = 33)	Non-DCD (n = 30)	*p*	Total (n = 63)	OR (95% CI)	*p*
Environmental						
Mother’s age at infant’s birth M (SD)	28.39 (7.11)	30.80 (5.70)	0.146	29.54 (6.54)	1.061 (0.981–1.153)	0.147
Father’s age at infant’s birth M (SD)	32.82 (8.74)	33.72 (8.56)	0.544	33.25 (8.58)	1.020 (0.958–1.088)	0.538
Family income (BRL) M (SD)	2012.85 (1144.62)	2843.52 (1673.67)	**0.013**	2412.21 (1470.28)	1.001 (1.000–1.001)	**0.016**
Mother’s formal education N (%)						
Less than High School	18 (54.5)	15 (50.0)		33 (52.4)		
High School	11 (33.3)	8 (26.7)	0.560	19 (30.2)	0.476 (0.107–1.890)	0.488
College	4 (12.1)	7 (23.3)		11 (17.5)		
Father’s formal education N (%)						
Less than High Scholl	23 (69.7)	19 (63.3)	0.208	42 (66.7)		
Completed High School	10 (30.3)	8 (26.7)		18 (28.6)	--	--
College	--	3 (10.0)		3 (4.8)		
Siblings N (%)	14 (42.4)	9 (30)	0.436	23 (36.5)	1.1842 (0.397–9.973)	0.587
KIDI—parents’ knowledge M (SD)	0.60 (0.08)	0.63 (0.08)	0.142	0.61 (0.09)	99.063 (0.253–72,701.109)	0.134
IRS—Interaction Rating Scale M (SD)	53.57 (14.58)	54.50 (13.14)	0.793	54.01 (13.81)	1.005 (0.969–1.043)	0.789
AHEM—Home Affordances for Development M (SD)	54.57 (17.39)	50.00 (14.52)	0.405	52.76 (16.27)	0.982 (0.940–1.023)	0.389

Note: M: Mean; SD Standard Deviation; N: Number; significant results are presented in bold.

**Table 3:** Multivariate logistic regression model for DCD.

Risk Factors	B	Exponential (B)	Wald *X*^2^	*p*	OR (95% CI)	R^2^	*p*	Hosmer- Lemeshow
Sex male	2.44	11.472	9.76	**0.002**	11.47 (2.48–53.02)	0.524	**0.006**	0.929
Ventilatory support	−0.006	0.99	0.062	0.803	0.994 (0.95–1.04)			
BPD	3.11	22.54	4.75	**0.029**	22.54 (1.37–370.61)			
Family Income	0.001	1.00	6.86	**0.009**	101(1.00–1.00)			

Note. BPD: Bronchopulmonary dysplasia; significant results are presented in bold.

## Data Availability

The data presented in this study are available on request from the corresponding author due to ethical reasons.

## Abbreviations

The following abbreviations are used in this manuscript:

DCD	Developmental Coordination Disorder
